# Sidt2 is a key protein in the autophagy-lysosomal degradation pathway and is essential for the maintenance of kidney structure and filtration function

**DOI:** 10.1038/s41419-021-04453-6

**Published:** 2021-12-18

**Authors:** Meng-ya Geng, Lizhuo Wang, Ying-ying Song, Jing Gu, Xin Hu, Cheng Yuan, Meng Yang, Wen-jun Pei, Yao Zhang, Jia-lin Gao

**Affiliations:** 1 Department of Endocrinology and Genetic Metabolism, The First Affiliated Hospital of Wannan Medical College (Yijishan Hospital of Wannan Medical College), Wuhu, 241001 People’s Republic of China; 2 Institute of Endocrine and Metabolic Diseases, Department of Endocrinology and Genetic Metabolism, The First Affiliated Hospital of Wannan Medical College (Yijishan Hospital of Wannan Medical College), Wuhu, 241001 People’s Republic of China; 3 School of Clinical Medicine, Wannan Medical College, Wuhu, 241002 People’s Republic of China; 4 Anhui Province Key Laboratory of Biological Macro-molecules Research (Wannan Medical College), Wannan Medical College, Wuhu, 241002 People’s Republic of China; 5 Department of Biochemistry and Molecular Biology, Wannan Medical Collage, Wuhu, 241002 People’s Republic of China

**Keywords:** Macroautophagy, Kidney diseases

## Abstract

The regulation and homeostasis of autophagy are essential for maintaining organ morphology and function. As a lysosomal membrane protein, the effect of *Sidt2* on kidney structure and renal autophagy is still unknown. In this study, we found that the kidneys of *Sidt2*^*−/−*^ mice showed changes in basement membrane thickening, foot process fusion, and mitochondrial swelling, suggesting that the structure of the kidney was damaged. Increased urine protein at 24 h indicated that the kidney function was also damaged. At the same time, the absence of *Sidt2* caused a decrease in the number of acidic lysosomes, a decrease in acid hydrolase activity and expression in the lysosome, and an increase of pH in the lysosome, suggesting that lysosomal function was impaired after *Sidt2* deletion. The accumulation of autophagolysosomes, increased LC3-II and P62 protein levels, and decreased P62 mRNA levels indicated that the absence of the *Sidt2* gene caused abnormal autophagy pathway flow. Chloroquine experiment, immunofluorescence autophagosome, and lysosome fusion assay, and Ad-mcherry-GFP-LC3B further indicated that, after *Sidt2* deletion, the production of autophagosomes did not increase, but the fusion of autophagosomes and lysosomes and the degradation of autophagolysosomes were impaired. When incubating *Sidt2*^*−/−*^ cells with the autophagy activator rapamycin, we found that it could activate autophagy, which manifested as an increase in autophagosomes, but it could not improve autophagolysosome degradation. Meanwhile, it further illustrated that the *Sidt2* gene plays an important role in the smooth progress of autophagolysosome processes. In summary, the absence of the *Sidt2* gene caused impaired lysosome function and a decreased number of acidic lysosomes, leading to formation and degradation disorders of the autophagolysosomes, which eventually manifested as abnormal kidney structure and function. *Sidt2* is essential in maintaining the normal function of the lysosomes and the physiological stability of the kidneys.

## Introduction

The traditional view is that lysosomes are the cells’ garbage disposals to remove wastes produced by cells [1–3]. However, many studies have shown that the role of lysosomes is far more complex, and may involve cell signal transduction, tumorigenesis, development, and other aspects that affect the life activities of the body [4–10]. Lysosomes are usually enriched in tissues such as the liver and kidney [11]. Therefore, lysosomal dysfunction is also closely related to tissue diseases of the liver and kidney, such as Gaucher’s disease [12], mucopolysaccharidosis [13], Niemann-Pick disease [14], delayed glomerulosclerosis [15], idiopathic membranous nephropathy [16], etc. Lysosomal membrane proteins (LMPs) are the membrane components whose function is not only to maintain the integrity of the lysosome, but which are also involved in various aspects such as intracellular signal transduction and regulation that are essential for maintaining the function of the lysosome and cell life activities [17–19].

To date, more than 100 LMPs have been discovered, but the functions of most of them are still unknown [20]. Transmembrane 7 superfamily member 1 (TM7SF1) is essential for the maintenance of renal podocyte function [21] and plays an important role in the process of kidney development [22]. Chloride Voltage-Gated Channel 5 (ClC-5) is a chloride ion (Cl(^−^)) channel expressed in renal tubules, which is essential for normal renal tubular function [23]; when it is mutated, it may cause Dent’s disease [24]. Overexpression of Chloride Voltage-Gated Channel 7 (ClC-7) prevents the apoptosis of renal tubular epithelial cells caused by impaired redox state [25]. These studies demonstrate that, as components of lysosome-enriched kidney tissue, LMPs are essential to maintain normal function, but the specific mechanism of their related pathogenicity is still unclear.

SID1 transmembrane family, member 2 (Sidt2) is a newly discovered LMP that is highly expressed in liver and kidney tissues [26]. Previous studies have shown that *Sidt2* deletion can cause liver-related diseases, which manifest as liver steatosis and liver lipid metabolism disorders [11, 18]. In a recent study, we found that the kidneys of *Sidt2*^*−/−*^mice also experienced damage, but the specific mechanism is unclear. Lysosomes are important executive organelles for autophagy [10]. Is autophagy regulation involved? In this study, we examined the correlation between lysosomal function (autophagy) and disease, and to explore the underlying mechanism involving *Sidt2* that causes kidney damage, which will be of great help for the study of the correlation between LMP and disease.

## Results

### *Sidt2*^*−/−*^ model shows that kidney damage is associated with autophagolysosome accumulation

**Fig. 1:**
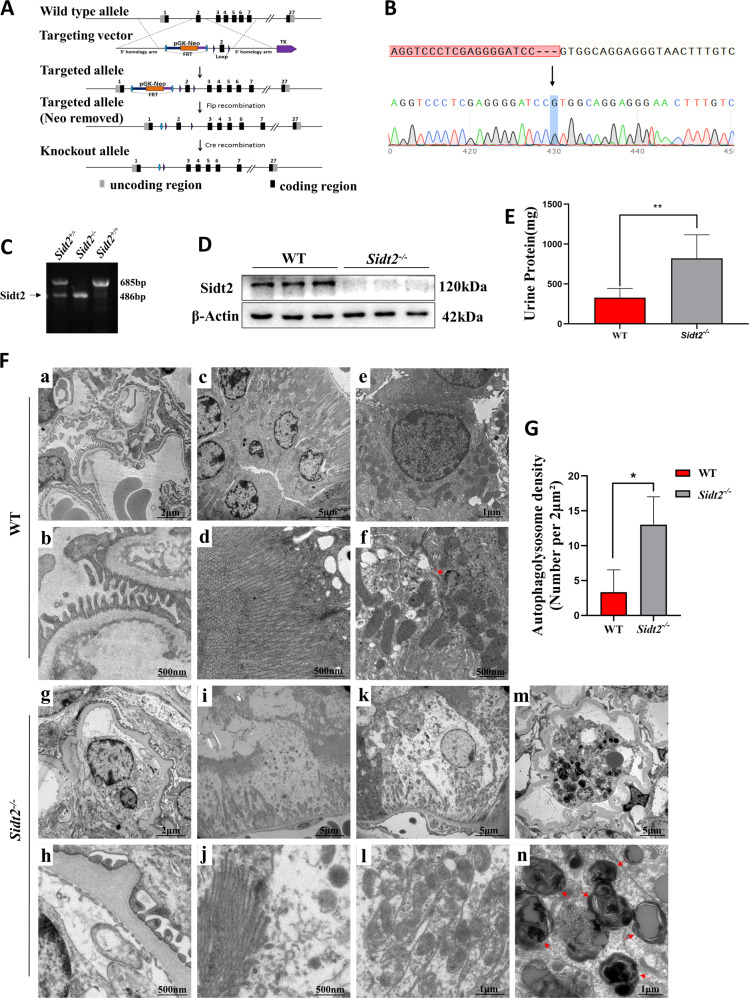
Kidney damage and autophagolysosome accumulation in *Sidt2*^*−/−*^ mice. **A** Cre-LoxP system gene targeting schematic; **B** above is the exported sequence diagram of *Sidt2* gene knockout mouse tail DNA; below is the sequencing map, the arrow indicating the location of the missing gene. Compared with WT mice, a 199 bp gene loss occurs in exon 2; **C** DNA level verification of *Sidt2* (extracted from tail tissue). The primer-amplified product contains the base knockout region, shown as *Sidt2*^*+ /+*^(WT), *Sidt2*^*+/−*^, or *Sidt2*^*−/−*^; **D** protein level verification to detect Sidt2 protein expression levels by western blot; **E** kidney 24 h urine protein in WT and *Sidt2*^*−/−*^ mice; **F** ultra-micro-morphological structure of the kidneys of WT mice (a, f) and *Sidt2*^*−/−*^ mice (g–n). Compared with the WT mouse, the *Sidt2*^*−/−*^ mouse kidney displays foot process fusion, basement membrane thickening (g, h), renal tubular epithelial cell edema, microvilli damage (i, j), mitochondrial destruction (k, l), and autophagolysosome accumulation (m, n); **G** total number of renal autophagolysosomes in WT and *Sidt2*^*−/−*^ mice. *P < 0.05, **P < 0.01.

The method of constructing *Sidt2*^*−/−*^ mice is shown in Fig. 1A. The obtained homozygous mouse tail DNA was sequenced and the sequencing result was unimodal (Fig. 1B), and a 199 bp fragment loss occurred in the second exon. Through PCR genotype identification, we found that *Sidt2*^*+/+*^(Wild Type, WT) mice had 685 bp DNA segments, whereas *Sidt2*^*−/−*^ mice had 486 bp (Fig. 1C). As shown in Fig. 1D, the Sidt2 protein could hardly be detected in *Sidt2*^*−/−*^ mice than that in WT, which suggested that the model was successfully constructed. By urine test, it was found that the 24 h-urine protein was significant increased in *Sidt2*^*−/−*^ mice compared with WT (Fig. 1E), suggesting that the kidney filtration barrier was impaired after the *Sidt2* deleted. Transmission electron microscopy observations (Fig. 1F) showed that, compared with WT mice (a–f), *Sidt2*^*−/−*^ mouse kidneys exhibited diffuse fusion of foot processes, thickening of the glomerular basement membrane (g, h), renal tubular epithelial cell edema, microvilli damage (i, j), mitochondrial edema, vacuole-like changes, and the disappearance of spines (k, l). Interestingly, *Sidt2*^*−/−*^ mice have also exhibited a large number of autophagolysosome accumulations (shown by red arrows, m, n), and the number were significantly higher than that of the control (Fig. 1G). At the cellular level, we found that *Sidt2* was also essential for the survival of kidney cells. The deletion of the *Sidt2* gene causes decreased proliferation and increased apoptosis of MPC5 and SV40 MES 13 cells (Supplementary Fig. 1).

### *Sidt2* gene deletion leads to changes in the number and function of acidic lysosomes in mouse kidney cells

**Fig. 2:**
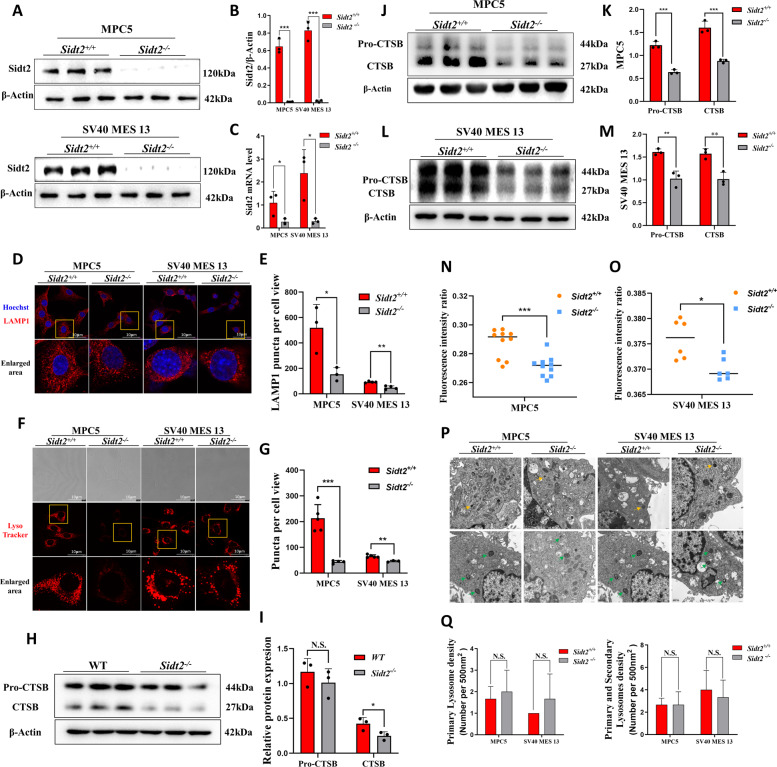
Effect of *Sidt2* deletion on the total number of lysosomes, the number of acid lysosomes, and the lysosomal environment and acid hydrolase. **A** Sidt2 protein expression level determination by western blot; **B** statistical charts of the western blot test results; **C** *Sidt2* mRNA expression level determination by qRT-PCR; **D** LAMP1 immunofluorescence in MPC5 and SV40 MES 13 cells before and after *Sidt2* knockout; **E** statistical chart of (**D**); **F** LysoTracker determination of the number of functional lysosomes before and after *Sidt2* knockout in MPC5 and SV40 MES 13 cells; **G** statistical graph of the LysoTracker results; **H** Cathepsin B expression in WT and *Sidt2*^*−/−*^ mice kidney; **I** statistical chart of (**H**); **J** Cathepsin B expression in MPC5 cells before and after *Sidt2* knockout; **K** statistical chart of (**J**); **L** Cathepsin B expression in SV40 MES 13 cells before and after *Sidt2* knockout; **M** statistical chart of (**L**); **N** LysoSensor detection of the lysosomal pH change on *Sidt2* knockout in MPC5 cells; **O** LysoSensor detection of the lysosomal pH change on *Sidt2* knockout in SV40 MES 13 cells; **P** electron microscopy application to observe the change in the number of lysosomes in kidney cells on *Sidt2* knockout (yellow arrows mark primary lysosomes and green arrows mark secondary lysosomes); **Q** statistical chart of the (**P**). *P < 0.05, **P < 0.01, ***P < 0.001.

Crispr-Cas9 technology was used to knock out the *Sidt2* gene in MPC5 and SV40 MES 13 cells to obtain the cell models. The mRNA and protein level verifications (Fig. 2A–C) were performed by qRT-PCR and western blotting respectively, which show that the models were successfully constructed. The lysosome-mediated degradation system is a key step in autophagy degradation. As an indispensable LMP, will the knockout of the *Sidt2* affect the expressions of lysosome-related proteins? We measured the major LMP lysosomal-associated membrane protein 1(LAMP1). The results showed that the expression of LAMP1 decreased after *Sidt2* deletion in MPC5 and SV40 MES 13 cells (Fig. 2D, E). We further used LysoTracker to label the acidic lysosomes, and found that the number of acidic lysosomes decreased after the *Sidt2* deletion in both types of cells (Fig. 2F, G). Subsequently, we measured the lysosomal cathepsin B (CTSB) and found that in mouse kidney tissue (Fig. 2H, I), MPC5 cells (Fig. 2J, K) and SV40 MES 13 cells (Fig. 2L, M), the expressions of CTSB in the *Sidt2*^*−/−*^ models were all reduced, indicating that the proteolytic enzyme activity in the lysosomes was decreased when the *Sidt2* gene was deleted. The samely, the expression of the precursor CTSB in the two types of cells of the *Sidt2*^*−/−*^ group was significantly reduced, indicating that the CTSB was also affected after *Sidt2* deletion. We further used LysoSensor to detect the acidic environment of the lysosomes. The lower the fluorescence intensity ratio, the higher the pH value in the lysosome. The results showed that after *Sidt2* deletion, the pH value increased (Fig. 2N, O) and the acidification was abnormal. In order to further explore whether the above lysosomal abnormalities were due to abnormal numbers of lysosomes or abnormal lysosomal functions, we investigated the cells by electron microscopy (Fig. 2P). We found that there are no obvious changes in the number of primary lysosomes and the total number of lysosomes (including primary and secondary lysosomes) after *Sidt2* deletion (Fig. 2Q).

### Renal autophagy pathway is abnormal after *Sidt2* removal

**Fig. 3:**
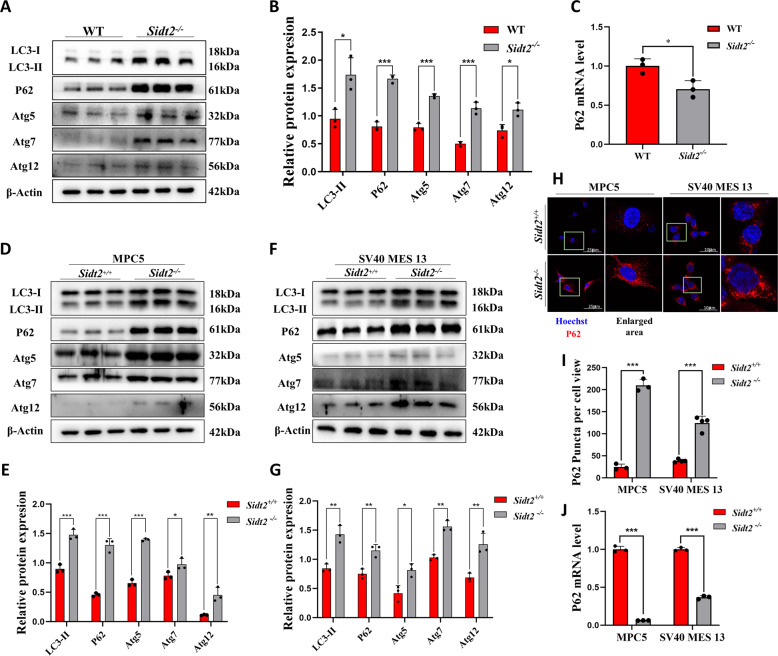
*Sidt2* deletion disrupts the autophagy pathway. **A** Detection of WT and *Sidt2*^*−/−*^ cell autophagy-related protein expression levels by western blot; **B** statistical charts of the western blot test results; **C** *P62* mRNA expression level in kidney tissues of WT and *Sidt2*^*−/−*^ mice; **D** expression levels of MPC5 cell autophagy pathway proteins before and after *Sidt2* knockout; **E** statistical graph of (**D**) chart; **F** expression levels of autophagy pathway proteins in SV40 MES 13 cells before and after *Sidt2* knockout; **G** statistical chart of the (**F**) chart; **H** P62 immunofluorescence in MPC5 and SV40 MES 13 cells before and after *Sidt2* knockout; **I** statistical charts of the immunofluorescence results; **J** P62 mRNA expression levels in MPC5 and SV40 MES 13 cells before and after *Sidt2* knockout. *P < 0.05, **P < 0.01, ***P < 0.001.

As mentioned above, a large number of autophagolysosome accumulations were observed in the kidney cells of *Sidt2*^*−/−*^ mice, suggesting that the autophagy pathway was abnormal. Using western blot analysis, it was shown that there was a significant increase in protein level of LC3-phosphatidylethanolamine conjugate (LC3-II) and Sequestosome 1(P62) in kidney of *Sidt2*^*−/−*^ mice, and an increase in autophagy-related protein expressions of Autophagy-related 5(Atg5), Autophagy-related 7(Atg7), and Autophagy-related 12(Atg12) (Fig. 3A, B). However, the P62 mRNA level was significantly decreased (Fig. 3C), suggesting that the autophagy pathway was abnormal after the elimination of *Sidt2* in vivo. Then we found that the expressions of the autophagy proteins LC3-II, P62, Atg5, Atg7, and Atg12 were also increased (Fig. 3D–G) in the two types of kidney cells after *Sidt2* deletion, which was consistent with the in vivo. The increase in LC3-II indicated an increase in autophagosomes in the kidney cells after *Sidt2* deletion, which may be caused by the activation of autophagy or the blocked degradation of autophagosomes. P62 immunofluorescence (Fig. 3H) clearly shown an increase in P62 content in the *Sidt2*^*−/−*^ cells (Fig. 3I). Contrary to the protein level, qRT-PCR showed that the P62 decreased significantly at the mRNA level (Fig. 3J), indicating that the increase in P62 was related to insufficient degradation. The increases in Atg5, Atg7, and Atg12 indicated that autophagy began and formed normally, but the accumulation of P62 also suggested autophagy process obstacles, and this contradiction required us to further explore the true situation of autophagy flux.

### In vitro chloroquine application indicates reduced autophagy flux after *Sidt2* loss

**Fig. 4:**
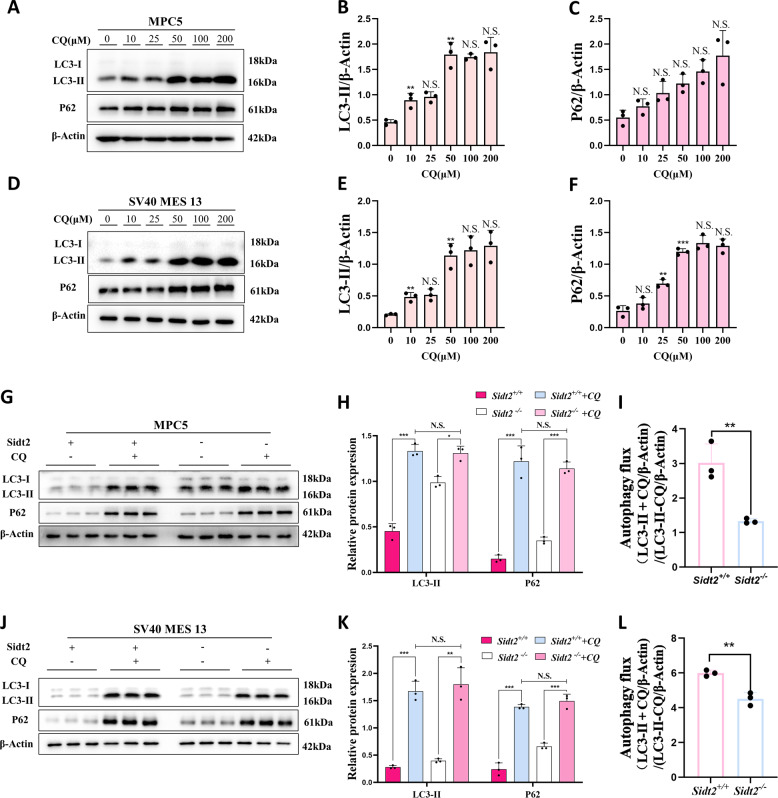
Autophagy degradation disorder after *Sidt2* deletion in mouse kidney cells. **A** LC3-II and P62 expression by MPC5 cells incubated for 16 h with 0, 10, 25, 50, 100, or 200 μM CQ shown by western blot; **B** LC3-II statistical chart of MPC5 cells; **C** P62 statistical chart of MPC5 cells; **D** SV40 MES 13 cell incubation for 16 h with 0, 10, 25, 50, 100, or 200 μM CQ. Western blot shows LC3-II and P62 expression; **E** LC3-II statistical chart of SV40 MES 13 cells; **F** SV40 MES 13 cell P62 statistical chart; **G** determination whether CQ induces the expression of key autophagy proteins before and after *Sidt2* knockout in MPC5 cells; **H** statistical chart of (**G**); **I** changes in the autophagy flux in MPC5 cells on *Sidt2* knockout (LC3-II + CQ/β-Actin)/(LC3-II − CQ/β-Actin) (ref. [27]); **J** determination whether chloroquine induces the expression of key autophagy proteins before and after *Sidt2* knockout in SV40 MES 13 cells; **K** statistical chart of (**J**); **L** changes of the autophagy flux in SV40 MES 13 cells on *Sidt2* knockout (LC3-II + CQ/β-Actin)/(LC3-II − CQ/β-Actin) (ref. [27]). *P < 0.05, **P < 0.01, ***P < 0.001.

The chloroquine experiment is one of the classic experiments for observing autophagy flux. In order to understand the reasons for the increases in LC3-II and P62 after *Sidt2* deletion, we used chloroquine (CQ), a downstream inhibitor of autophagy, to act on the cells to observe the autophagy flux. First, we used different concentration gradients to confirm that the saturation concentration inhibited by CQ was 50 μM (Fig. 4A–F) in the MPC5 and SV40 MES 13 cells. On top of this concentration, when the CQ concentration continued to increase, LC3-II and P62 did not increase further to reach saturation. Therefore, we used 50 μM CQ stimulation over 16 h to completely inhibit autophagy flux, and at this time, the LC3-II differences between the *Sidt2*^*+/+*^group and *Sidt2*^*−/−*^ group disappeared in the MPC5 and SV40 MES 13 cells. Similarly, after 50 μM CQ processing, the P62 differences caused by the deletion of *Sidt2* also disappeared (Fig. 4G, H, J, K). The statistics on autophagy flux showed that it decreased when *Sidt2* was missing (Fig. 4I, L), which further confirmed that the increases in LC3-II and P62 expression after the *Sidt2* deletion were due to the failure of autophagy clearance rather than the increasing of the level in autophagy (the activation of autophagy).

### Disruption of autophagosome-lysosome fusion in vitro after *Sidt2* deletion

**Fig. 5:**
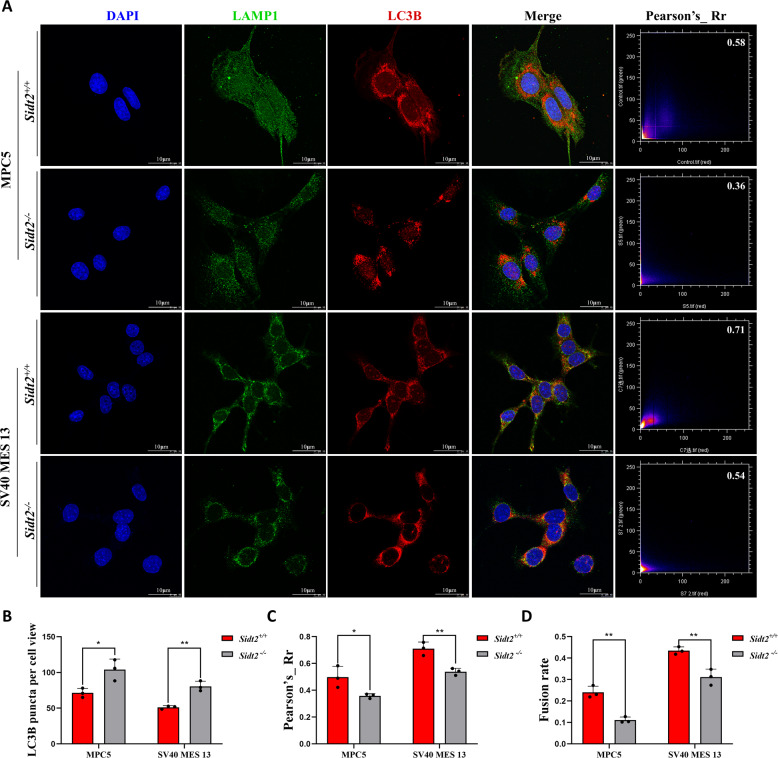
Autophagosome and lysosome fusion is prevented after *Sidt2* deletion. **A** Immunofluorescence co-localization of LC3B and LAMP1 in MPC5 and SV40 MES 13 cells before and after *Sidt2* knockout, analysis with Pearson’s correlation coefficient; **B** comparison of LC3B fluorescence points before and after *Sidt2* knockout in MPC5 and SV40 MES 13 cells; **C** comparison of Pearson’s correlation coefficient before and after *Sidt2* knockout in MPC5 and SV40 MES 13 cells; **D** comparison of the fusion rate before and after *Sidt2* knockout in MPC5 and SV40 MES 13 cells (the ratio of the number of co-localized fluorescent spots of LAMP1 and LC3B to the number of fluorescent spots of LAMP1); *P < 0.05, **P < 0.01.

Clearance and degradation are the middle and late stages in autophagy process, involving the autophagosomes fusion with lysosomes to form autophagolysosomes and the degradation of autophagolysosomes. In order to further explore the reasons for the failure of autophagy clearance after *Sidt2* deletion, we first studied the fusion process of autophagosomes and lysosomes. The red fluorescence used to mark LC3B can represent autophagosomes, and LAMP1 labeled with green fluorescence as the lysosome marker. At the basic autophagy level, we investigated the immunofluorescence colocalization of LC3B and LAMP1 after *Sidt2* deletion in MPC5 and SV40 MES 13 cells (Fig. 5A). The results showed that after *Sidt2* deletion, the number of LC3B fluorescence spots increased (Fig. 5B) and the Pearson correlation coefficient between LC3B and LAMP1 decreased (Fig. 5C), suggesting decreased co-localization. In order to exclude the possibility that the decrease in the co-localization with LC3B was caused by a decrease in the number of LAMP1 fluorescence points after the *Sidt2* deletion, we further standardized the fusion rate of autophagosomes and lysosomes. We calculated the ratio of the number of fluorescent spots co-localized with LAMP1 and LC3B to the number of fluorescent spots of LAMP1 in the two groups of cells after the *Sidt2* deletion. The results showed that the fusion rate decreased after the deletion of the *Sidt2* (Fig. 5D), indicating that the fusion of autophagosomes and lysosomes was impaired.

### Ad-mCherry-GFP-LC3B fluorescence double-labeling suggests that autophagolysosome formation and its degradation pathway were blocked after *Sidt2* deletion

**Fig. 6:**
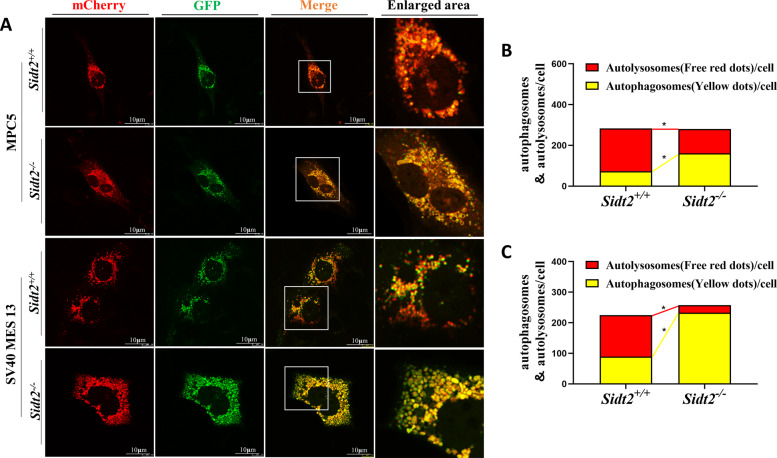
Autophagy lysosomal degradation pathway is blocked after *Sidt2* deletion. **A** Ad-mCherry-GFP-LC3B application to detect autophagy flow in MPC5 and SV40 MES 13 cells before and after *Sidt2* knockout; **B** statistical chart of MPC5 cell autophagosome and autophagolysosome detection results; **C** statistical chart of SV40 MES 13 cell autophagosome and autophagolysosome detection results. *P < 0.05.

The Ad-mCherry-GFP-LC3B transfection experiment can be used to detect changes in dynamic autophagy flux within cells. During the process of autophagy, mCherry-GFP-LC3B gathers on the autophagosome membrane and manifests itself in the form of yellow spots under a fluorescence microscope. When autophagosomes and lysosomes are fused to form autophagolysosomes, the acidic environment within the lysosome will quench the fluorescence of GFP so that it manifests itself in the form of red spots. Therefore, GFP bound to LC3 can only be used to detect autophagosomes, while mCherry can detect autophagosomes and autophagolysosomes at the same time. When the green and red fluorescence spots are combined and displayed as yellow fluorescence spots, this corresponds to autophagosomes. At this time, the red fluorescence can indicate autophagolysosomes, and it can also indicate the smoothness of autophagolysosome formation [27]. MPC5 cells and SV40 MES 13 cells in both the *Sidt2*^*+/+*^ and *Sidt2*^*−/−*^ groups were transfected with Ad-mCherry-GFP-LC3B adenovirus and photographed with a confocal laser microscope (Fig. 6A). It was found that the number of yellow fluorescent dots increased and the number of red fluorescent dots decreased (Fig. 6B, C) after *Sidt2* deletion, shown that the number of autophagosomes increased and the autophagolysosomes decreased in the *Sidt2*^*−/−*^ group. This is because there were obstacles to autophagolysosome formation, and the autophagolysosome degradation pathways were blocked, which led to autophagosome degradation obstacles.

### Rapamycin did not improve the P62 accumulation caused by *Sidt2* deletion but rather exacerbated it

**Fig. 7:**
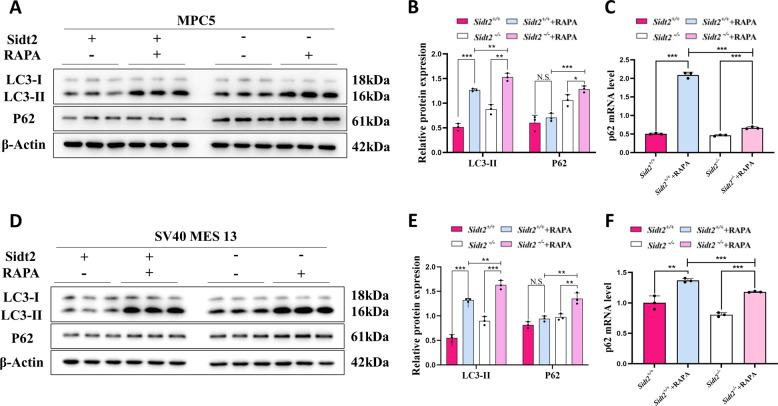
Upstream activator of autophagy cannot improve P62 accumulation induced by *Sidt2* knockout. **A** Changes of key autophagy proteins in MPC5 cells on adding rapamycin; **B** statistical chart of (**A**); **C** *P62* mRNA expression in MPC5 cells before and after adding rapamycin; **D** changes of key autophagy proteins in SV40 MES 13 cells on adding rapamycin; **E** statistical chart of (**D**); **F** *P62* mRNA expression in SV40 MES 13 cells on adding rapamycin; *P < 0.05, **P < 0.01, ***P < 0.001.

Rapamycin (RAPA) is an inhibitor of mTOR and activates autophagy. When RAPA acted on MPC5 and SV40 MES 13 cells of the *Sidt2*^*+/+*^and *Sidt2*^*−/−*^ groups, it increased the amounts of LC3-II in both groups. This meant that RAPA could activate the upstream pathway of autophagy. The samely, we found that the increase in LC3-II level in the *Sidt2*^*−/−*^ group with RAPA was more obvious than that in control (*Sidt2*^*+/+*^group), showing that when RAPA acted on *Sidt2*^*−/−*^ group, the degradation of autophagosomes still was not improved. Meanwhile, we also observed the changes in P62 and found that when RAPA acted on the *Sidt2*^*+/+*^ group of MPC5 and SV40 MES 13 cells, P62 did not change significantly, indicating that the autophagy flux was normal. When RAPA acted on the *Sidt2*^*−/−*^ group, the expression level of P62 was further increased compared to that before administration, and was also more obvious than that in control after RAPA treatment (Fig. 7A, B, D, E). Subsequently, in order to explore the reasons for the inconsistent expression of P62 protein after the *Sidt2*^*+/+*^ and the *Sidt2*^*−/−*^ group were treated with RAPA, we measured the mRNA levels of P62 and found that the level increased after RAPA treatment in both the *Sidt2*^*+/+*^ and the *Sidt2*^*−/−*^ groups, and the *Sidt2*^*+/+*^ group had a higher level than that in the *Sidt2*^*−/−*^ group (Fig. 7C, F), which could further prove the autophagy flow was smooth in the *Sidt2*^*+/+*^ group, while the autophagy flow disorder occurred at the end of autophagy in the *Sidt2*^*−/−*^ group, that is, the autophagolysosomes degradation link. We also studied the effect of Sidt2 on the proliferation and apoptosis of kidney cells under the condition of activated autophagy, and found that after serum-free medium induced cell activation of autophagy, compared with the control group, the proliferation of the *Sidt2*^*−/−*^ group was further inhibited, and the apoptosis was more obvious (Supplementary Fig. 2).

## Discussion

A large number of studies have shown that LMPs are involved in kidney function or structural homeostasis [21–25]. Our previous study found that the LMP Sidt2 affects the inflammatory signaling pathway and causes damage to the mouse glomerular mesangial cells [28]. In this study, for the first time, we investigated the effects on the structure and function of mouse kidney caused by the LMP Sidt2. It was found that after *Sidt2* deletion led to the fusion of the mouse kidney foot processes, thickening of the basement membrane, renal tubular epithelial cell edema, microvilli damage, and increased proteinuria at 24 h, indicating that the loss of *Sidt2* leads to impaired filtration function and structure of the mouse kidney, but its detailed mechanism is not clear.

In view of the fact that LMPs are important functional units of the lysosome [17] and lysosomal dysfunction is one of the common pathogenic factors of many chronic kidney diseases (CDKs) (refs. [4, 29]), we have carried out related investigations on lysosomes after *Sidt2* deletion and found that the number of acidic lysosomes decreased and the pH of the lysosomes increased in vitro. However, there were no obvious changes in the numbers of primary lysosomes bound to autophagosomes and the total lysosomes by electron microscopy analysis. Cathepsins represent the largest group of proteolytic enzymes in lysosomes [30], among which CTSB is one of the most abundant lysosomal proteases [31]. Since cathepsins need to be acidified for maturation (activation), a change in pH will eventually lead to a significant reduction in protein degradation [32]. In vivo and in vitro levels showed decreased activity and content of lysosomal acid hydrolase, further confirming that *Sidt2* inhibits lysosomal function by affecting the maturation of proteases in the lysosomes.

Meanwhile, the lysosome acts as a recycling center for degrading cellular metabolites and waste products at the end of autophagy [1]. Normal lysosomal degradation function is essential for autophagy, maintaining cell homeostasis, and enabling cells to survive in physiological states [33–36]. The occurrence and development of many kidney diseases have been proved to be related to abnormal autophagy [37–39]. Recent studies have found that Sidt2 is a lysosomal DNA/RNA transporter. The unconventional types of autophagy through RNautophagy and DNautophagy (RDA) are important for selective RNA/DNA degradation [40]. We have previously found that the deletion of the *Sidt2* damages the fusion of autophagosomes and lysosomes, causing damaged mitochondria to be unable to be degraded, which results in damage to the structure and function of skeletal muscle [41]. Therefore, in this study, we explored autophagy when kidney damage was caused by *Sidt2* deletion. We observed that autophagolysosomes were accumulated in the kidneys of *Sidt2*^*−/−*^ mice through electron microscopy. Autophagy is a dynamic process, including the formation of autophagosomes and the formation and degradation of autophagolysosomes. The formation of early autophagosomes is controlled by the Atg gene, and the formation of LC3-II is a sign of autophagosome formation [42]. We found that the expressions of autophagy-related proteins Atg and LC3-II both increased after *Sidt2* deletion, indicating that the autophagy activity may be enhanced. However, the accumulation of LC3-II may also be caused by blockage of the autophagolysosomes pathway at the end of autophagy.

In order to further explore the effect of *Sidt2* on the renal autophagy state, the expression and formation of P62 cargo protein were measured, and also the effects of drugs that target autophagy (CQ and RAPA) were investigated. P62 needs to be combined with LC3B and then degraded in the lysosome. An increase in the level of P62 protein often represents a blockage of the autophagy flux [43]. In order to eliminate the cause of the increase in P62 production, we measured the mRNA level of P62, and found that the production of P62 was reduced, so the accumulation of LC3-II was probably due to hindered degradation. In order to further explore the process of autophagy, we conducted a chloroquine flip experiment. Chloroquine can neutralize the acidic environment of the lysosome, and subsequently, destroy the function of the lysosome and inhibit autophagy [44]. The increase in LC3-II protein before and after CQ treatment reflects the amount of autophagosomes degraded by lysosomes, which can reflect the activity of autophagy. The same CQ flip test can also be used to determine the amount of P62 degradation through the autophagolysosome pathway. Consistent with our results, the deletion of the *Sidt2* led to a reduction in autophagy flow due to the blocked end of autophagy. At the same time, in the RAPA experiment, LC3-II and P62 protein levels increased significantly in the *Sidt2*^*−/−*^ group, which can further support that the lack of *Sidt2* impairs the end phase of autophagy.

The end phase of autophagy includes the formation and degradation of autophagolysosomes. We used LAMP1 and LC3B immunofluorescence co-localization experiments to detect the fusion of autophagosomes and lysosomes [45, 46], and the results showed that the fusion of autophagosomes and lysosomes was blocked after *Sidt2* deletion. The Ad-mCherry-GFP-LC3B double-labeling experiment detects the formation and degradation of autophagolysosomes [27]. It further confirmed that the blockage of the formation and degradation of autophagolysosomes is the main reason for the damage to kidney structure and function after *Sidt2* deletion.

**Fig. 8:**
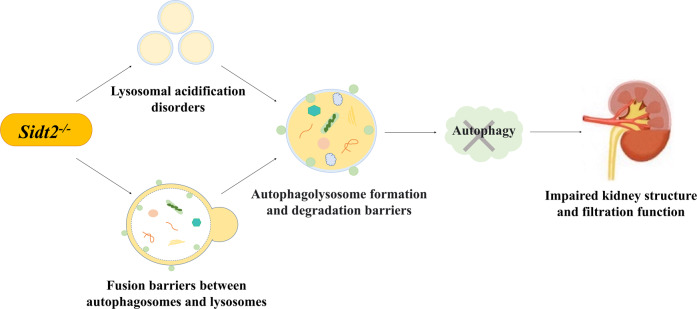
*Sidt2* deletion causes renal autophagy, structure, and function impairments. The deletion of Sidt2 gene leads to lysosomal acidification disorders, and the fusion of autophagosomes and lysosomes is blocked, which leads to the formation and degradation barriers of autophagolysosomes. Impaired autophagy ultimately leads to impaired kidney structure and filtration function.

From the above results, we can conclude that the absence of the *Sidt2* causes a decrease in the number of acidic lysosomes, and that the acidification dysfunction of the lysosomes affects the maturation of hydrolyzed enzymes. This may be the most important cause of damage to the autophagy lysosome degradation pathway in addition to autophagosome and lysosome fusion disorders. Meanwhile, the impaired autophagy process may be closely related to the damaged renal structure and filter function after *Sidt2* deletion (Fig. 8). Our study only addresses part of the effects of *Sidt2* on kidney structure and function, but what is interesting is that the accumulation of key autophagy proteins and the impairment of kidney structure and function caused by *Sidt2* deletion are similar to the changes in key autophagy proteins and significantly increased proteinuria seen in diabetic nephropathy [37, 47]. The repair of lysosome function may be a potential new strategy for the treatment of diabetic nephropathy [37]. We expect that *Sidt2* will provide a way to explore the pathogenesis and treatment of kidney diseases in the future.

## Materials and methods

### Experimental animals

Mouse models were constructed using the Cre-LoxP system in the same way as mentioned previously [48] and were edited by the Shanghai Model Organisms Center (please refer to the Supplementary Materials for detailed methods). All animal experiment programs were approved by the Animal Council of Wannan Medical College and all of their recommendations were followed.

### Urine protein testing in mice

Randomly selected 12-week-old WT mice and *Sidt2*^*−/−*^ male mice were placed in metabolic cages and fasted but given water. 24 h urine samples were collected, and then tested for urine protein (Nanjing Jiancheng Bioengineering Institute, CHN, C035-2-1) (please refer to the Supplementary Materials for detailed methods).

### Cells

MPC5 mouse glomerular podocytes were purchased from BeNa Culture Collection, and cultured with 10% FBS (Excell Bio, South America, FSP500) and low-glucose DMEM(Sigma-Aldrich, 6046). SV40 MES 13 mouse glomerular mesangial cells were purchased from the typical culture preservation committee cell bank of the Chinese Academy of Sciences, and cultured with 10% FBS (Excell Bio, South America, FSP500) and high-glucose DMEM (Sigma-Aldrich, 6429). The CO_2_ concentration of the cell culture box was maintained at 5% and the temperature was maintained at 37 °C. Chloroquine (CQ) and rapamycin (RAPA) were purchased from Sigma-Aldrich (C6628, V900930). 10 mM CQ and 25 mg/mL rapamycin were both dissolved in ultra-pure water. CQ was added to the petri dish so that the final concentration was 50 μM, and incubated for 16 h as an autophagy inhibitor. Rapamycin was added to the petri dish at a final concentration of 15 μM and incubated for 2 h as an autophagy activator.

### CRISPR/Cas9 gene-editing lentivirus vector system was used to remove the *Sidt2* gene

Lenticrispr-v2 (AddgenePlasmid49535, Feng Zhang) plasmids were purchased, and the sgRNA online design tool (http://crispr.mit.edu/) was used to design sgRNA targeting *Sidt2*. The primer sequences were as follows: F: 5′-ACCACACCGTGACCC CAC-3′, R: 5′-GTTGCGGGTCACTGGTGT-3′, synthesized by biotechnology company (Sangon Biotech, Shanghai, CHN). Using the CRISPR/Cas9 lentivirus system, the Lenticrispr-v2 plasmid was linearized and linked to the annealed sgRNA duplex to construct the Lenticrispr-v2-Sidt2 recombinant plasmid. Vigofect (Vigorous Biotechnology, Beijing, CHN) transfection reagent was used to transfect the Lenticrispr-v2 plasmid and Lenticrispr-v2-Sidt2 recombinant plasmid in order to construct the lentivirus. The obtained lentivirus was transfected into the MPC5 cells and SV40 MES 13 cells. After 48 h, the cells were incubated with purinomycin for 72 h to selection. The surviving cells were the *Sidt2*^*+/+*^ group and *Sidt2*^*−/−*^ group that had been successfully transfected.

### RNA isolation and real-time PCR assay

Trizol (Invitrogen) was used to extract RNA from mouse kidney cells, and the specific RT-PCR method was as described previously [21]. The primers sequences of RT-PCR were as follows: Actin (sense: F: 5′- GGACTCCTATGTGGGTGACG-3′, R:5′-CTTCTCCATGTCGTCCCAGT-3′), P62(sense: F:5′-GGACCCATCTACAGAGGCT G-3′, R: 5′-ATCACAATGGTGGAGGGTGC-3′), Sidt2(sense: F:5′-TAGTGCCTGTTACCACGTCTGC-3′, R:5’-GGATGCAGTCTGTGTAGAGCACA-3′).

### Western blotting

The specific method is as mentioned before [28]. In this study, primary antibodies included rabbit anti-LC3B (1:1000; Sigma-Aldrich L7543), rabbit anti-P62 (1:1000; Abcam ab205719), mouse anti-β-Actin (1:1000; Sigma-Aldrich A5316), rabbit anti-Sidt2 (1:1000; Invitrogen PA5-69064), rabbit anti-Atg5 (1:1000; Cell Signaling Technology 9980S), rabbit anti-Atg7 (1:1000; Cell Signaling Technology 8558S), rabbit anti-Atg12 (1:1000; Cell Signaling Technology 2011S), mouse anti-LAMP1 (1:1000; Santa Cruz Biotechnology H4A3), rabbit anti-cathepsin B (1:1000; Cell Signaling Technology 31718S).

### Ad-mCherry-GFP-LC3B

The cells were inoculated on Nunc glass-bottomed Petri dishes. After growing to a suitable density, Ad-mCherry-GFP-LC3B was added (Beyotime, Shanghai, CHN, C3011-10 mL), resulting in a final concentration of 10−11 vp/mL. After stimulating for 48 h, the numbers of mCherry and GFP fluorescence points were directly observed under the laser confocal microscope (Leica SP8, Germany).

### Immunofluorescence

The cells were inoculated on glass-bottomed Petri dishes and fixed with paraformaldehyde. After blocking, the cells were incubated overnight with primary antibody, and then incubated with secondary antibody for 90 min in the dark. The cells were photographed using the laser scanning confocal microscope (Leica SP8, Germany) after dyeing the nuclei with DAPI (1 μg/mL). Primary antibodies included P62 (1:200; Abcam ab205719), LAMP1 (1:200; Santa Cruz Biotechnology H4A3), LC3B (1:200; Sigma-Aldrich L7543); secondary antibodies included anti-mouse secondary anti Cy3-conjugated Affinipure goat anti-mouse IgG (H + L) (1:200; Proteintech SA00009-1), CoraLite488-conjugated Affinipure goat anti-mouse IgG (H + L) (1:200; Proteintech SA00013-1), CoraLite594-conjugated Affinipure goat anti-rabbit IgG (H + L) (1:200; Proteintech SA00013-4). For LysoTracker (Beyotime, Shanghai, CHN, C1046), we also inoculated the cells on glass-bottomed Petri dishes. After growing to a suitable density, cell culture fluid containing a LysoTracker final concentration of 75 nM was added to the cells, which were photographed after 45 m of stimulation. Use ImageJ to count the number of fluorescent dots and the Pearson correlation coefficient.

### Transmission electron microscope observations

From each group, 3−5 12-week-old *Sidt2*^*−/−*^ and WT male mice were randomly selected to prepare electron microscopy samples of the kidney tissue, and the specific method is as described earlier [48], randomly take pictures of the sections using an electron microscope, there are more than 10 pictures taken from kidney sections for each mouse, randomly select 3–5 pictures of 8000× magnification from the electron microscope pictures of each mouse, calculate the number of autophagolysosomes in the picture, and compare the *Sidt2*^*−/−*^ group with the WT group. Regarding the preparation of cell electron microscope samples, scrape no less than 10^5^ cells with a cell scraper and collect them in an EP tube, then centrifuge at 800 rpm/min for 5 m, discard the supernatant and fill it with the electron microscope fixative. After being stored in a 4° refrigerator for more than 1 h, it can be submitted for inspection. The electron microscope shot was taken by KingMed diagnostics (Guangzhou, China).

### LysoSensor

The cells were inoculated on a 96-well plate. After growing to a suitable density, preheated (37 °C) medium containing 1 µM LysoSensorDND-160^®^ (Thermo Fisher L7545) was added. The cells were incubated for 5 min at 37 °C, and then the medium was removed and replaced after washing the cells three times with PBS. WellScan mode was used to read the plate in BioTek Citation 5 (BioTek, Winooski, VT, USA). Fluorescence was measured using Ex360nm/Em450nm and Ex380nm/Em540nm excitation/emission wavelengths, and the lysosome pH was determined from the ratio of Em540 to Em450.

### Statistical analysis

The results were expressed as the mean ± SEM. Statistical comparison between two groups was performed using unpaired t-tests. When Graphics 8.0 software was used for statistical analysis, *P* < 0.05 was considered statistically significant. Each group of experiments in this study was repeated at least three times independently.

## Supplementary information

Supplementary materials

## Data Availability

The datasets generated and/or analyzed during the current study are available from the corresponding author on reasonable request.
