# Supplementary material for: Sidt2 is a key protein in the autophagy-lysosomal degradation pathway and is essential for the maintenance of kidney structure and filtration function
Source: Cell Death Dis. 2021 Dec 18;13(1):7. doi: 10.1038/s41419-021-04453-6 (PMC8684554; doi:10.1038/s41419-021-04453-6)
Supplement: Supplementary file 1 — Supplementary materials [file 41419_2021_4453_MOESM1_ESM.docx]

**Supplementary materials**

**Materials and methods**

**Experimental animals**

The Cre-LoxP system was used to construct *Sidt2^-/-^* mice, and the construction was commissioned to Shanghai Model Organisms Center. The ES cell targeting vector was constructed by the ET-clone method, and the design of targeting vector is shown in the figure (Figure 1A). Insert the FRT site between the first exon and the second exon, insert the neo resistance in the site, and insert the LoxP site across the second exon. The vector was linearized, electrotransfected into ES cells, and positive cells were obtained after screening with G418 and Ganc drugs. After identification by long-segment PCR, the positive ES cells were cloned and amplified and injected into the blastocysts of C57BL/6J mice to obtain chimeric mice. Mate chimeric mice with FLP mice to obtain de-neo-resistant mice, and *Sidt2* was eliminated by Dppa3-Cre mice. *Sidt2* heterozygous mice were obtained by mating with C57BL/6J mice, and homozygous mice were obtained by mating heterozygous mice. All mice were kept at standard temperature and humidity in the SPF animal room, maintained a 12-hour light/dark cycle, and fed with standard food without restricting their drinking water. In animal studies, compared gender and age-matched WT mice with *Sidt2^-/-^* mice. The experiments used 8-12 week old mice for the study. All animal experiment programs were approved by the Animal Council of Wannan Medical College and all of their recommendations were followed.

**Urine protein testing in mice**

WT mice and *Sidt2^-/-^* mice aged 12 weeks were randomly selected and placed in metabolic cage, fasted but given water, and urine was collected for 24h. Use urine protein determination kit (Nanjing Jiancheng Bioengineering Institute, CHN, C035-2-1). First, configure the CBB application liquid according to the method of CBB: double- distilled water 1:4. Set up the blank tube, standard tube, and measuring tube. Add 3ml of CBB application application liquid to each tube, 0.05ml of double-distilled water into the blank tube, 0.05ml of 563mg/L protein standard solution into the standard tube, and 0.05ml sample into the measuring tube. After mixing well, add it to a 96-well plate, and each sample is set with 6 or more secondary wells. Let it stand for 5 minutes then put it into the microplate reader. Set the wavelength to 595nm, the light path to 1cm. Adjust the zero with double- distilled water, and measure the absorbance value of each well. $Urine protein concentration (mg/L)＝\frac{（measured OD value-blank OD value）}{（standard OD value-blank OD value）}*standard substance concentration (563mg/L)$

**Annexin V-FITC/PI Apoptosis Flow Cytometer**

Use Annexin V-FITC/PI Double Stain Apoptosis Detection Kit (KeyGEN BioTECH, Nanjing, CHN, KGA108) to detect the degree of programmed cell death by flow cytometry (Beckman Coulter Cytoflex). After 8 hours of incubation in medium with or without serum, MPC5 and SV40 MES 13 cells were harvested and washed twice with PBS. The cells were centrifuged at 2000 rpm/min for 5 minutes, and stained with Annexin V-FITC and PI in the dark, and then the cells were counted.

**Cell proliferation detection**

Using Cell-Light EdU Apollo 567 (RiboBio, Guangzhou, CHN, C10310-1) kit, cells were incubated for 2 hours at 37°C in a working solution containing 50 μM 5-ethynyl-2'-deoxyuridine (EdU). Carry out the relevant steps to detect EdU signal according to the manufacturer's instructions. Shoot under a fluorescent inverted microscope (Olympus, Janpan), and use ImageJ for statistics.

**Supplementary Figure 1**


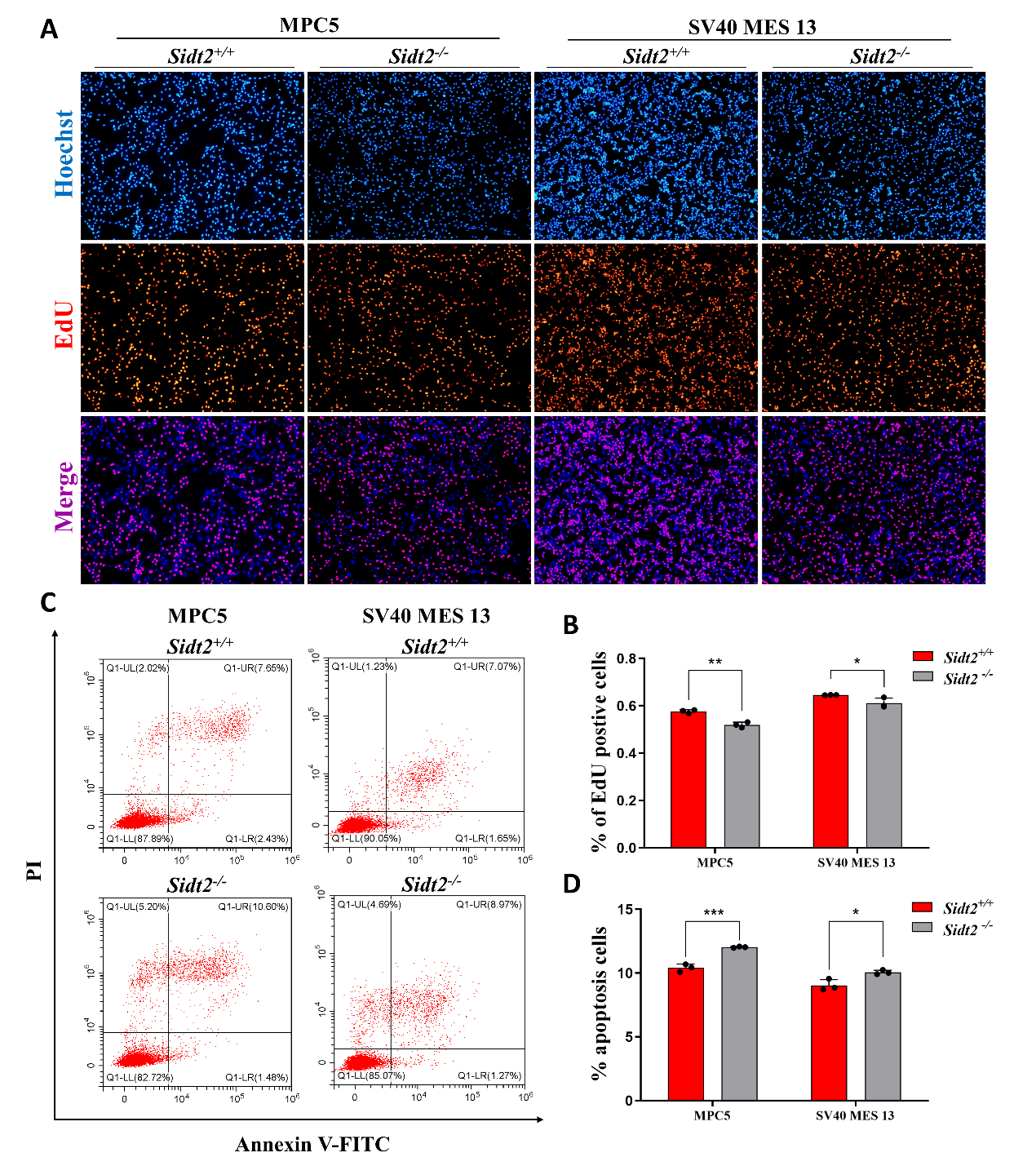


**Supplementary Figure 1** **Effects of *Sidt2* gene deletion on the proliferation and apoptosis of mouse kidney cells**

**(A)** Proliferation of MPC5 and SV40 MES 13 cells before and after *Sidt2* knockout;

**(B)** Statistical chart of graph **A**; **(C)** Apoptosis of MPC5 and SV40 MES 13 cells before and after *Sidt2* knockout; **(D)** Statistical chart of graph **C**; **P*<0.05, ***P*<0.01, ****P*<0.001.

**Supplementary Figure 2**


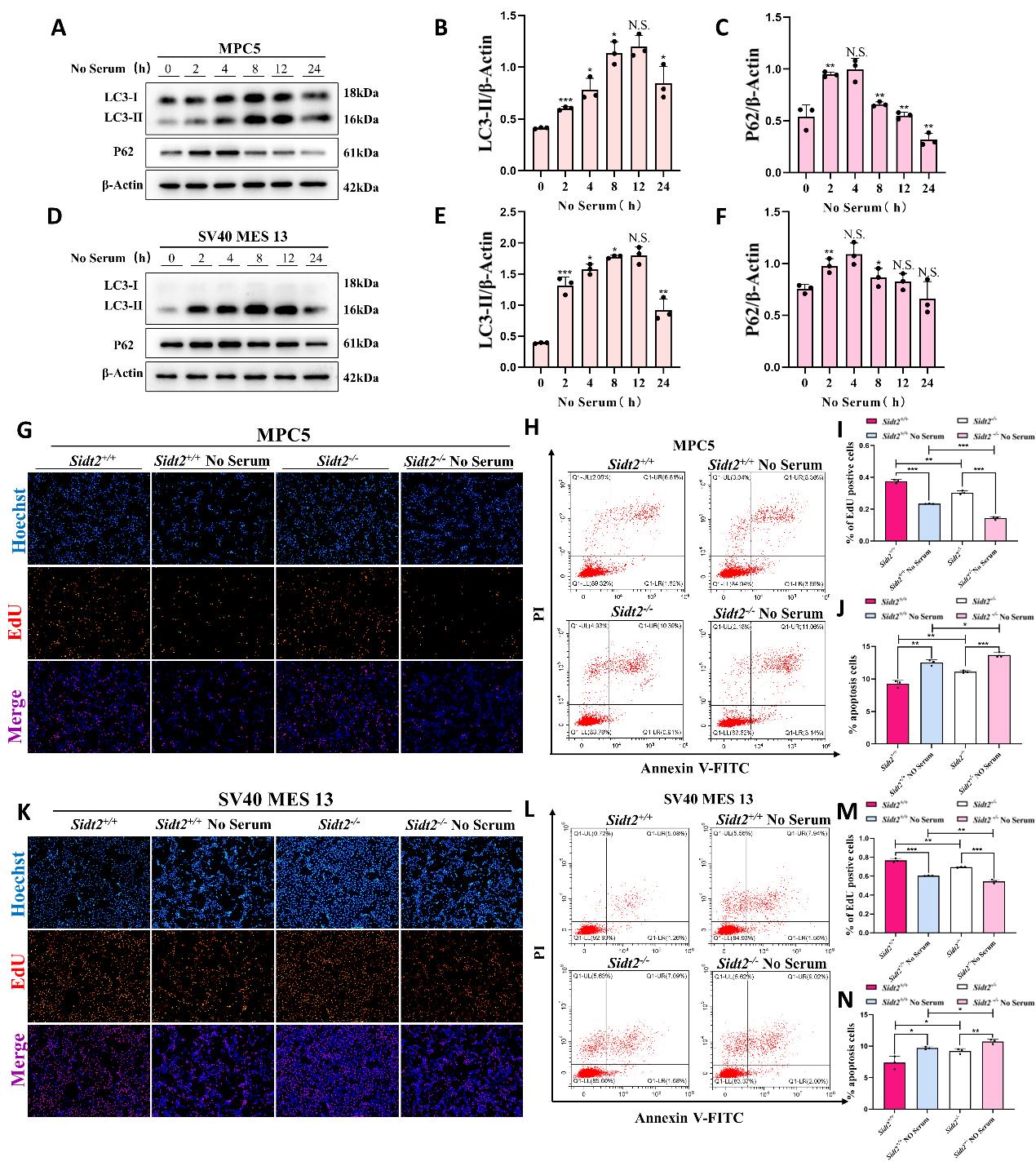


**Supplementary Figure 2 Effects of *Sidt2* gene deletion on the proliferation and apoptosis of mouse kidney cells under serum-free induction.**

**(A)** LC3-II and P62 expression in normal MPC5 cells after serum-free induction at different times; **(B)** LC3-II expression statistical chart of graph **A**; **(C)** P62 expression statistical chart of graph **A**; **(D)** LC3-II and P62 expression in normal SV40 MES 13 cells after serum-free induction at different times; **(E)** LC3-II expression statistical chart of graph **D**; **(F)** P62 expression statistical chart of graph **D**; **(G****)** Serum-free induction of cell proliferation in MPC5 cells before and after *Sidt2* knockout; **(H)** Serum-free induction of apoptosis in MPC5 cells before and after *Sidt2* knockout; **(I)** Cell proliferation statistical chart of graph **G**; **(J)** Cell apoptosis statistical chart of graph **H**; **(K)** Serum-free induction of cell proliferation in SV40 MES 13 cells before and after *Sidt2* knockout; **(L)** Serum-free induction of apoptosis in SV40 MES 13 cells before and after *Sidt2* knockout; **(M)** Cell proliferation statistical chart of graph **K**; **(N)** Cell apoptosis statistical chart of graph **L**; **P*<0.05, ***P*<0.01, ****P*<0.001.
